# Kinetics of Biological Removal of the Selected Micropollutants and Their Effect on Activated Sludge Biomass

**DOI:** 10.1007/s11270-018-4015-7

**Published:** 2018-10-25

**Authors:** Ewa Liwarska-Bizukojc, Małgorzata Galamon, Przemysław Bernat

**Affiliations:** 1 Institute of Environmental Engineering and Building Installations, Lodz University of Technology, Al. Politechniki 6, 90-924 Lodz, Poland; 2 Faculty of Biology and Environmental Protection, Department of Industrial Microbiology and Biotechnology, University of Lodz, ul. Banacha 12/16, 90-237 Lodz, Poland

**Keywords:** Activated sludge, Acclimation, Biodegradation, Inhibition, Kinetic parameters, Micropollutants

## Abstract

17α-Ethinylestradiol (EE2), diclofenac (DCF), and 4-nonylphenol (4NP) belong to the most common micropollutants (MPs) occurring in municipal wastewater treatment plants (WWTPs). The WWTPs are the primary barrier against the spread of micropollutants in the environment. The aim of this work was to study the kinetics of biological removal of the three aforementioned micropollutants from wastewater and to check whether the acclimation of biomass influenced on the kinetic parameters. In addition, the effect of MPs on the biochemical activity of microorganisms was tested. DCF inhibited the respiration activity of biomass to the highest extent, followed by 4NP and EE2, respectively. DCF occurred to be less susceptible to microbial decomposition than the other two MPs and was removed from wastewater at the lowest degree of 58%. The degrees of removal of EE2 and 4NP were higher than that of DCF and equal to 93 and 71%, respectively. The kinetic parameters determined in this work can be used in modelling and simulation of the removal of micropollutants from wastewater. They improve the predictive ability of the biokinetic models. The acclimation of the biomass to the relevant micropollutant does not influence on the kinetic parameters of biomass growth; however, it causes the increase of the yield coefficient for heterotrophic biomass.

## Introduction

Micropollutants occur in the wastewater treatment plant (WWTPs) influents usually in the concentration range between 0.1 and 10 μg L^−1^ (Luo et al. 2014). The term “micropollutants” comprises the wide range of such chemicals as pharmaceuticals, pesticides, heavy metals, and synthetic surfactants. Many of them are endocrine-disrupting compounds (EDCs).

Steroid hormones also belong to the group of micropollutants, and their concentrations in wastewater usually do not exceed 1 μg L^−1^. A major contributor to the total estrogenicity of municipal wastewater is one of the steroid hormones, a synthetic oestrogen-17α-ethinylestradiol (EE2), which is widely used in the contraceptive pills. It was estimated that about 40% of the total EE2 used by one person, i.e., about 10.5 μg day^−1^, reached the sewage influent (Hamid and Eskicioglu 2012). Many non-steroidal synthetic endocrine-disrupting compounds like some pesticides and personal care products (PCP) also reach wastewater treatment plants with municipal wastewater. Among them the highest concentrations in the raw municipal wastewater were reported for nonylphenol and diclofenac. For nonylphenol, the observed levels exceeded 100 μg L^−1^ (Terzić et al. 2008; Janex-Habibi et al. 2009; Rosal et al. 2010; Ruel et al. 2010). At the same time, the reported concentrations of diclofenac varied from below 0.001 to 94.2 μg L^−1^ (Kasprzyk-Hordern et al. 2009; Stamatis et al. 2010; Behera et al. 2011; Ruel et al. 2012; Loos et al. 2013; Gao et al. 2014). The concentration of pesticides, particularly atrazine, and fire retardant tris (2-chloroethyl) phosphate (TCEP) were not as high and typically did not exceed 1 μg L^−1^ in the raw municipal wastewater (Reemtsma et al. 2008; Ruel et al. 2010; Campo et al. 2013; Loos et al. 2013; Stamatis and Konstantinou 2013).

The occurrence of the broad variety of micropollutants in the municipal wastewater made WWTPs the primary barriers against their spread in the environment, particularly in its aquatic compartment. In most of the WWTPs, activated sludge systems are applied as biological part of wastewater treatment. Thus, the effect of the micropollutants on activated sludge organisms and the efficiency of these treatment systems in the removal of the micropollutants is of the highest importance.

The degrees of removal of endocrine-disrupting compounds from wastewater measured in 14 countries and regions were in the range from 12.5 to 100% (Luo et al. 2014). According to the classification proposed by Luo et al. (2014), human estrogens (E1, E2, and E3), EE2, bisphenol A, and triclosan were highly removed from wastewater (degree above 70%); nonylphenol could be classified as moderately removed (degree from 40 to 70%), while atrazine and diclofenac were poorly removed (degree below 40%). At the same time, it was observed that bisphenol A, a highly water soluble compound, was almost completely removed in all tested WWTPs (Froehner et al. 2011), whereas the removal of the estrogenic hormones (E1, E2, and EE2), being hydrophobic compounds, was lower and varied between 70 and 87% (Froehner et al. 2011). It was estimated that on average, 80% of the load of micropollutants was removed by the conventional activated sludge systems (Ruel et al. 2012).

The possible removal pathways of the micropollutants from wastewater include volatilization, microbiological degradation, and sorption. The values of the Henry’s law constant determined for the steroid hormones indicate that the contribution of volatilization in the removal of these compounds from wastewater is negligible under normal pressure and temperature conditions. Volatilization can be the relevant process only for some treatment systems with large surface area, e.g., waste stabilization ponds (Clouzot et al. 2013). Globally, volatilization concerned only volatile micropollutants but the limit of volatility was rarely and not clearly indicated (Pomiès et al. 2013). Sorption of EDCs onto suspended colloids and particles contributed to the removal of these chemicals with waste sludge. In the literature, two-parameter semi-empirical models, i.e., Langmuir or Freundlich isotherms, were most commonly used with regard to EDCs (Clouzot et al. 2013; Pomiès et al. 2013). In some works, their authors assumed that sorption was instantaneous due to the fact that this process was usually faster than biodegradation (Pomiès et al. 2013). Thus, the microbiological decomposition of micropollutants plays one of the most important roles in their removal from wastewater and still requires thorough studies. What is more, the prediction of the fate of micropollutants in the activated sludge systems allows for the minimization of the presence of these compounds in the environment. It remains in agreement with water protection policy in European Union countries (European Commission 2013).

In this work, three micropollutants that commonly occur in the municipal wastewater in the European WWTPs were studied, namely 17α-ethinylestradiol (EE2), diclofenac (DCF), and 4-nonylphenol (4NP). Two of them, i.e., DCF and 4NP, were found at high concentrations at the level of 100 μg L^−1^ in the raw municipal wastewater and at the concentrations exceeding 1 μg L^−1^ in surface water. Although the removal of these micropollutants from wastewater has previously been studied, the knowledge about the kinetics of their biological decomposition is still in shortage. It concerned particularly the values of the kinetic parameters of biological removal of these micropollutants from wastewater.

The main objective of this work was to describe the kinetics of the biological treatment of wastewater containing the micropollutants, i.e., EE2, DCF, or 4NP, and to check the influence of the acclimation of biomass on the kinetic parameters. Additionally, the effect of micropollutants on the biochemical activity of mixed (activated sludge) and pure culture(s) of microorganisms was studied. The values of kinetic parameters determined in this study (with and without biomass acclimation) are going to be used in modelling and simulation of the removal of the micropollutants from wastewater, opening the door for the optimisation of the conditions of this process.

## Materials and Methods

### Tested Compounds

The following chemicals: 17α-ethinylestradiol (EE2), diclofenac (DCF), and 4-nonylphenol (4NP), qualified as micropollutants, were studied. They are able to interfere with the regular functioning of the endocrine system in humans and animals and thus are called EDCs. EE2 represents the estrogenic hormones, whereas DCF and 4NP belong to the non-steroidal estrogens. The choice of these micropollutants for this study was justified in Sect. 1. Chemical names and elemental composition of the studied compounds are as follows: EE2 for 17α-ethinylestradiol (C_20_H_24_O_2_), DCF for [2-(2,6-Dichloroanilin)phenyl]acetic acid (C_14_H_11_Cl_2_NO_2_), and 4NP for 4-(2,4-Dimethylheptan-3-yl)phenol (C_15_H_24_O).

### OECD Tests

The Closed Bottle Test OECD 301D and the Zahn-Wellens/EMPA test OECD 302B were applied in order to evaluate the biodegradability of the micropollutants tested. Both tests were performed according to the guidelines elaborated by OECD (OECD Chemical Group 1993a; b). Sodium dodecyl sulphate (SDS) was used as the reference substance in these OECD tests.

According to the guidelines included in the OECD 302B test, the contribution of sorption in the removal of the micropollutants in the activated sludge system was estimated. Due to the fact that sorption is a relatively rapid process, the values of chemical oxygen demand (COD) were determined after 3 h and compared with the results obtained at the beginning of the tests. On the basis of these results, the degree of sorption was calculated.

### ToxTrak™ Test

ToxTrak™ method is based on the reduction of resazurin, a redox-active dye, by the bacterial respiration (Method 10017 HACH, Loveland, CO, USA). The presence of any toxic substances in the sample decreases the rate of resazurin reduction, which can be measured colorimetrically. The endpoint of ToxTrak™ method is the inhibition of the bacterial respirometric activity. The results of this test (toxicity scores) were expressed as the degree of inhibition (DI) as a percentage (%) (HACH 2017).

In this work, ToxTrak™ test was used to measure the toxicity of micropollutants toward activated sludge microorganisms and the pure culture of bacteria *Escherichia coli* DSM 30083. The indigenous activated sludge biomass was taken from the second aeration chamber of the Zgierz WWTP. Each of the micropollutants was tested individually. The absorbance was measured with the use of spectrophotometer DR 6000 at *λ* = 603 nm. The test was made in accordance with the guidelines for ToxTrak™ (Method 10017 HACH, Loveland, CO, USA). Each sample was made in four replications. If necessary, the additional replications were made in order to obtain the reliable results.

### Dehydrogenase Activity Assay

Dehydrogenase (DHG) activity was determined with the use of 2,3,5-triphenyltetrazolium chloride (TTC) according to the procedure described by Liwarska-Bizukojc (2011) that adopted the approach previously proposed by Miksch (1985). The test consisted of the following stages: (1) addition of the reagents to the activated sludge in the following order: 0.2% solution of sodium sulphite (Na_2_SO_3_), distilled water (control sample) or the proper solution of the tested chemical (ionic liquid or methanol), and finally 1% solution of TTC; (2) incubation in the dark at the room temperature 21 °C ± 0.5; (3) sludge separation; (4) stopping the reaction by the addition of pure 100% methanol; (5) measurement of absorbance at 485 nm. The control sample was analysed in six replications, whereas the test sample in triplicate for each concentration studied. The results obtained for each concentration were averaged and then the degree of inhibition (*I*_DHG_) was calculated according to the following equation:

1 $$ {I}_{\mathrm{DHG}}=\frac{A_{\mathrm{c}}-{A}_{\mathrm{t}}}{A_{\mathrm{c}}}\cdot 100\left(\%\right) $$

where *A*_c_ is the mean value of dehydrogenase activity of activated sludge not exposed to the tested chemical (control sample) and *A*_t_ is the mean value of dehydrogenase activity of activated sludge exposed to the tested chemical (test sample).

The inhibition of DHG activity was studied in the range of concentration from 0.1 to 1000 μg L^−1^ of the micropollutant. Each of the micropollutants was tested individually.

### Oxygen Uptake Rate Tests

OUR tests were made in order to determine the kinetic parameters for the biological treatment of wastewater containing one of the micropollutants under study. They were conducted separately for each examined micropollutant. The chemical was dissolved in the synthetic wastewater at the concentration of 10 μg L^−1^. Also, the control tests without the addition of any micropollutants (only synthetic wastewater) were made. The composition of the synthetic municipal wastewater and the details about the OUR test methodology were presented elsewhere (Liwarska-Bizukojc et al. 2014). Each of the tests, including the control runs, was performed in three replicates.

The tests were carried out in a batch bioreactor of 4 l working volume equipped with the control and measurement devices at the constant temperature (20 °C). Wastewater with or without one of the micropollutants studied and activated sludge were introduced into the bioreactor and dissolved oxygen (DO) and oxygen uptake rate (OUR) were immediately measured. The biomass used in all OUR tests was acclimated to the substrate, i.e., synthetic municipal wastewater. In the selected OUR tests, the applied biomass was also acclimated to the relevant micropollutant. Thus, two types of OUR tests were made, i.e., the tests with the biomass acclimated and non-acclimated to the relevant micropollutant. Acclimation was conducted in the laboratory-activated sludge system described in the next subsection.

Based upon the results of the OUR tests, two parameters of Monod equation, i.e., the maximum specific growth rate of heterotrophic biomass (*μ*_max_) and the half saturation constant (*K*_S_) were determined. The value of *μ*_max_ was determined as the slope of OUR changes in time (*t*) during the exponential growth phase (Kappeler and Gujer 1992). The value of *K*_S_ was determined by the integration of the OUR curve in the range from the time, at which the specific growth rate (*μ*) is equal to the half of maximum specific growth rate (*μ*_max_) for activated sludge biomass to the end-time of the experiment, when OUR hardly changed in time. Additionally, the yield coefficient for the heterotrophic biomass (*Y*_H_) was estimated. It is defined as the ratio of biomass concentration change (expressed as volatile suspended solids) to substrate concentration change (expressed as COD soluble).

### Acclimation of Activated Sludge Biomass

Acclimation was conducted in the laboratory activated sludge system, to which the synthetic wastewater of the same composition as previously described (Liwarska-Bizukojc et al. 2014) was introduced. Laboratory-activated sludge system consisted of four aeration chambers and each of them was coupled with the individual clarifier. The working volume of the individual aeration chamber was 5.6 L. The influent was pumped continuously to each aeration chamber at the constant volumetric flow rate equal to 0.15 L h^−1^. Aeration flow rate was constant and identical in each experimental run (0.321 L_air_ L^−1^ min^−1^). The experiments were conducted at the ambient temperature (21 ± 1 °C).

Acclimation consisted of two stages. In the first stage, the synthetic wastewater without the micropollutant in the influent was added to the aeration chambers. COD of the influent and effluent were determined every 2 or 3 days in order to check the progress in the adaptation process (Xu et al. 2013). After 1 week of adaptation, the degree of COD removal was about 94% and remained on this level during the next days. It indicated that activated sludge biomass was acclimated to the composition of the synthetic municipal wastewater. Then, the second stage of acclimation started and different substrates were delivered to each chamber. To the first one, synthetic wastewater containing EE2 was added; to the second one, synthetic wastewater containing DCF; to the third one, synthetic wastewater containing 4NP; and finally, to the fourth one, only synthetic wastewater (control run). The concentration of each of the micropollutants tested was initially equal to 1 μg L^−1^ and later increased up to 10 μg L^−1^. In order to check the acclimation of activated sludge biomass, the degree of removal of each of the micropolutants studied was checked every 3 days. The stable values of the concentration of the micropollutants in the effluent indicated that the acclimation processes were finished. The degree of COD removal was at the level of 94 ± 1.5% during the second stage of acclimation processes.

### Analytical Methods

Volatile suspended solids (VSS), chemical oxygen demand (COD), and biochemical oxygen demand (BOD_5_) were determined in agreement with the standard procedures (APHA-AWWA-WEF 2012). The concentration of micropollutants was determined with the use of chromatographic techniques.

The quantitative determination of EE2 and DCF was made with the use of liquid chromatography system Agilent 1200 coupled with mass spectrometer QTRAP 4500 (Sciex). A Zorbax Eclipse XDB-C_18_ column (50 mm × 4.6 mm, 1.8 μm particle size) from Agilent was applied in these determinations. The mobile phase consisted of water (A) and methanol (B) with the addition of ammonium formate. The elution was made in the gradient mode. Initially, the ratio of A to B was 2:8; after 2 min, the mobile phase consisted of methanol only. Later on, within the next 4 min, the composition of the mobile phase returned to the starting point. Mobile phase flow rate was equal to 0.6 mL min^−1^. MS/MS analysis was performed in the MRM mode. For EE2, ion pair 295–145 was used, while for DCF, it was 293–249 at the negative polarization. The method was linear in the range from 0.05 to 20 ng mL^−1^. The concentration of 4-nonylphenol was determined with the help of gas chromatography system Agilent 7890A coupled with the mass spectrometer Agilent 5975c. The chromatographic separations were performed with the use of a DB 5 MS methyl polysiloxane (30 m × 0.25 mm id 0.25 mm ft) column. The initial temperature of the column was 60 °C and within 3 min, it increased at the constant rate of 20 °C min^−1^. The maximum temperature was 280 °C which was kept for 7 min. The carrier gas was helium flowing with the rate 1 mL min^−1^. The temperature of the injection port was 250 °C. The detection limit was 0.1 ng mL^−1^.

## Results and Discussion

### Biodegradability of the Micropollutants Tested

**Fig. 1:**
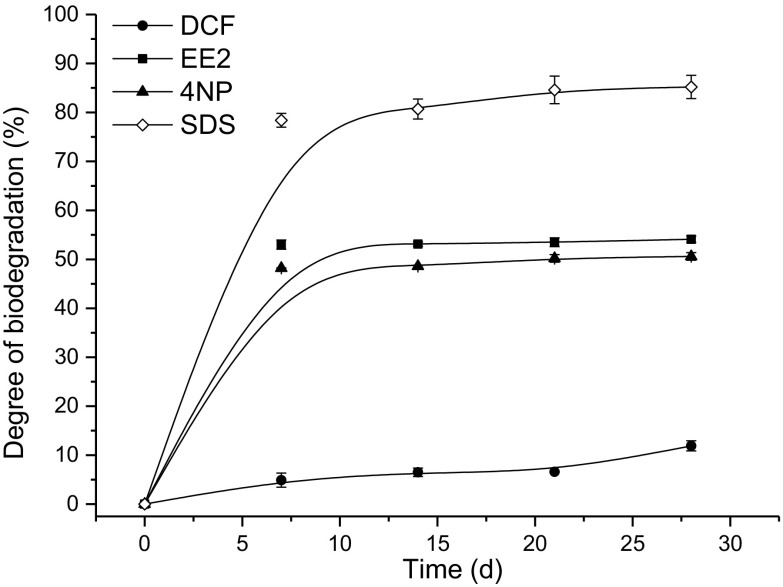
Changes of degree of biodegradation (results of the OECD test 301D)

The readily and inherent biodegradability of the micropollutants studied were evaluated upon the results of the OECD 301D test. They revealed that none of the micropollutants tested can be classified as readily biodegradable (Fig. 1). The pass level for readily biodegradability is 60% of theoretical oxygen demand (ThOD) according to the OECD guidelines (OECD Chemical Group 1993a; b). It should be noticed that two chemicals, i.e., EE2 and 4NP, were close to this level because they reached the degrees of degradation of 54.1 and 50.6%, respectively. The lowest degree of degradation was found for DCF (11.9%). At the same time, for the reference substance (SDS), the degree of degradation increased from 78.2 to 85.3% during the test (Fig. 1), what indicated that the test was run properly.

**Fig. 2:**
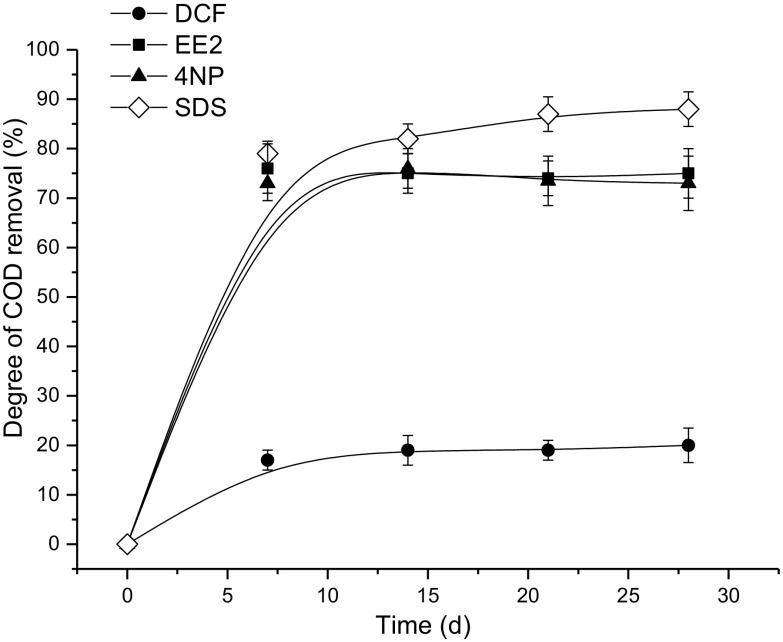
Changes of degree of COD removal (results of the OECD test 302B)

The results of the OECD 302B test were in agreement with those obtained in the OECD 301D test and showed that EE2 and 4NP were biodegradable, whereas DCF was less susceptible to the microbial decomposition. In the case of EE2 and 4NP, the degree of degradation exceeded the value of 70% during 14 days of test (Fig. 2). It was only slightly lower than the degree of degradation determined for the reference substance SDS, which was at the level of 82% (Fig. 2). The latter value proved the correctness of the performance of the OECD 302B test. According to the guidelines of this test, it is considered valid if the removal of COD in the reference samples is at least 70% within 14 days (OECD Chemical Group 1993a, b). The mean degree of sorption calculated on the basis of COD determination was the highest for 4NP (27.4 ± 2.3%). For the other two tested compounds, it was equal to 12.5 ± 1.7% for EE2 and 8.8 ± 1.1% for DCF. It indicated that the sorption played the most important role in the removal of 4NP. The compound of the lowest ability to be adsorbed onto sludge particles, namely DCF, occurred to be the least susceptible to biodegradation. In the case of the reference substance (SDS), the degree of sorption was 10.7 ± 0.8%.

### Effect of Micropollutants on Biochemical Activity of Activated Sludge Biomass

In order to evaluate the influence of the micropollutants on activated sludge microorganisms, two various assays were performed. These were dehydrogenase activity (DHG) assay and ToxTrak™ test. DHG assay was made towards mixed cultures (activated sludge) only, whereas ToxTrak™ test was conducted towards activated sludge microorganisms and the pure culture of bacteria *E. coli*.

**Fig. 3:**
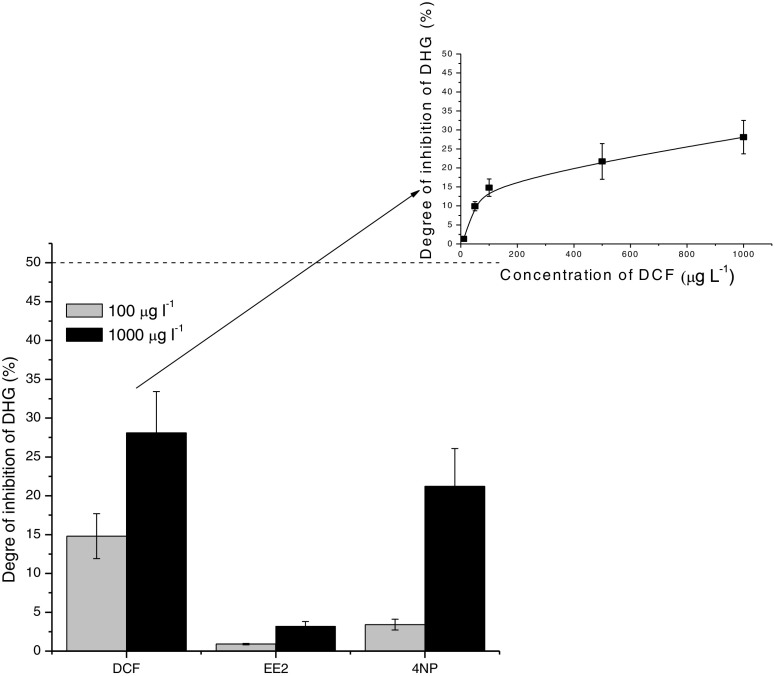
Effect of tested micropollutants on the dehydrogenase activity (DHG assay)

It occurred that EE2 and 4NP did not inhibit dehydrogenase activity at all, if their concentrations were not higher than 50 μg L^−1^, as the degrees of inhibition were below 3% (Fig. 3). Similarly, in the case of EE2, the degree of inhibition remained at the low level even at its concentration equal to 1000 μg L^−1^. DCF and 4NP exerted stronger effect on dehydrogenase activity than EE2 did in the range of concentrations between 50 and 1000 μg L^−1^, particularly at concentrations higher than 100 μg L^−1^ (Fig. 3). At the same time, none of the tested compounds reduced by half the biochemical activity of activated sludge microorganisms expressed as DHG activity (Fig. 3).

**Fig. 4:**
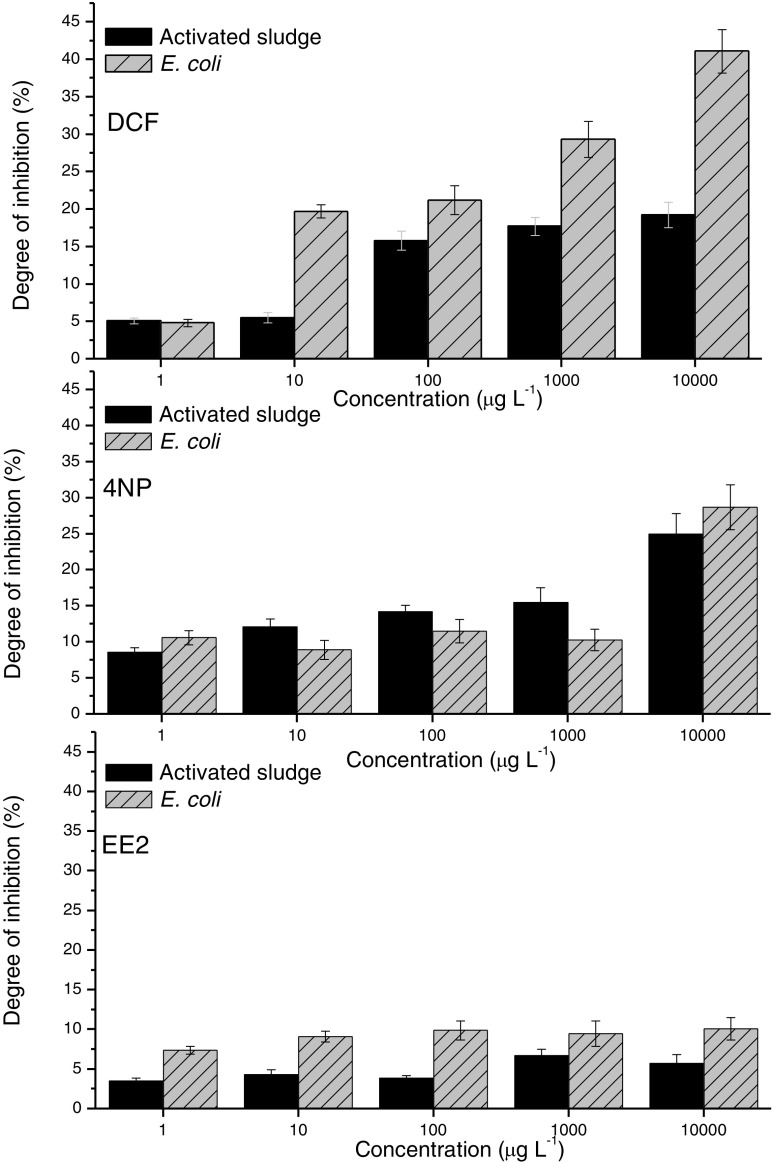
Effect of tested micropollutants on the respiration activity of microorganisms (ToxTrak™ test)

The results of ToxTrak™ tests were similar to those obtained in DHG assays, and they led to the same conclusions (Fig. 4). Out of all micropollutants studied, DCF inhibited the respiration activity of mixed cultures (activated sludge) as well as of pure culture (*E. coli*) to the highest extent. However, the effect of 4NP on biomass growth was stronger than that of EE2 irrespective of the type of biomass used in the test. Similarly, as it was found in DHG tests, none of the tested compounds reduced by half the respiration activity of microorganisms (neither in the mixed cultures nor in the pure culture). Generally, the micropollutants studied had stronger impact on *E. coli* culture compared with the mixed cultures of activated sludge. It confirmed that pure cultures of microorganisms were more sensitive than mixed cultures and thus the latter were usually used for the purpose of biological treatment of wastewater. Some exceptions from this rule were observed only in the case of EE2 (Fig. 4).

**Fig. 5:**
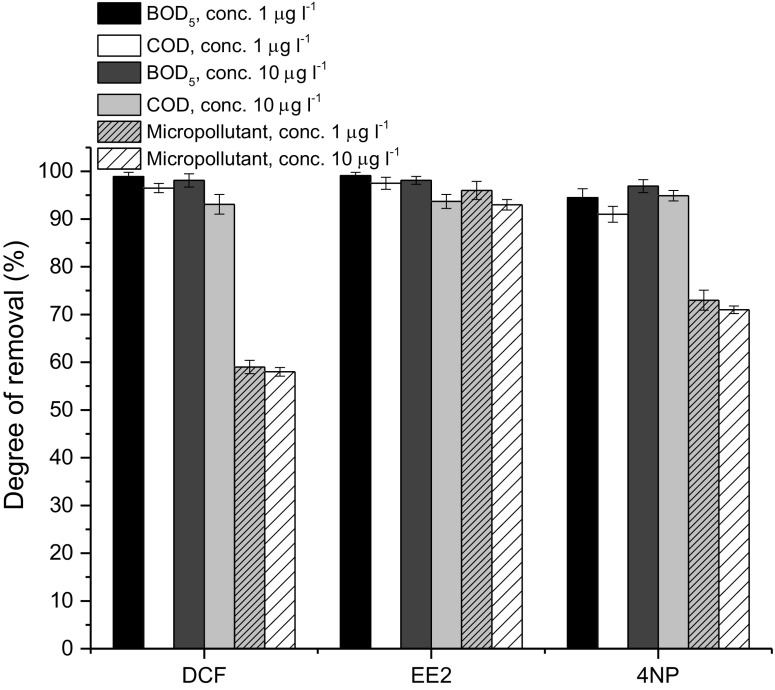
Removal of organic pollutants (BOD_5_, COD) and micropollutants tested during the acclimation tests

The results of respirometric tests were well correlated with the degrees of removal of the micropollutants from wastewater in the activated sludge system (acclimation tests). It occurred that DCF, being the substance that inhibited the respirometric activity to the highest extent out of all chemicals tested, was removed in the lowest degree from wastewater. Its degree of removal was at the level of 58%, while for EE2 and 4NP these degrees were higher than that and equal to 93 and 71%, respectively (Fig. 5).

### Kinetics of the Biological Treatment of Wastewater Containing Micropollutants

Kinetic parameters of Monod equation, i.e., *K*_S_ and *μ*_max_, were determined from the OUR tests. These tests were performed with the use of biomass acclimated to the synthetic wastewater (non-acclimated to the relevant micropollutant tested) and with the use of biomass acclimated to the synthetic wastewater with the relevant micropollutant. It allowed for the estimation of the effect of acclimation processes on the kinetics of removal of the studied micropollutants from wastewater.

The acclimation processes ran without disturbances. The degree of removal of organic compounds was high, and the acclimation experiments showed that the presence of micropollutants did not interfere with the efficient removal of organic pollutants. The degree of BOD_5_ removal was from 94.5 to 99.1%, whereas the degree of COD removal varied from 91 to 97.4%, depending on the tested micropollutant and its concentration (Fig. 5). What is more, the degrees of removal of micropollutants under study were here at similar levels in spite of the increase of their concentrations in the influent from 1 to 10 μg L^−1^ during the acclimation experiments (Fig. 5). The slight increase of degree of BOD_5_ and COD removal was found only in the case of 4NP most probably as a result of acclimation processes (Fig. 5). Similarly, Ferro-Orozco et al. (2013) found that the presence of bisphenol-A in the synthetic wastewater did not cause any negative effect on the biodegradation of the organic substrates.

The values of *K*_*S*_ determined in this study were in the range of half saturation constants found for biological treatment of municipal wastewater (Hauduc 2011). The presence of micropollutants in wastewater at 10 μg L^−1^ caused the decrease of the affinity of biomass to substrate. It was confirmed by higher values of *K*_S_ for each substrate containing one of the micropollutants tested than those in the control tests without the addition of micropollutants (Table 1). However, the increase of the values of *K*_S_ was not significant and did not exceed 68%, referring to the mean value of *K*_S_ determined in the control runs.

**Table 1:** Mean values of Monod equation parameters (*μ*_max_ and *K*_S_) and yield coefficient for heterotrophic organisms of activated sludge

	Mean *μ*_max_ (h^−1^)	Mean *K*_S_ (mg O_2_ L^−1^)	*Y*_XS_ (mg VSS mg COD^−1^)
Control	0.293 ± 0.038	13.52 ± 2.44	0.226 ± 0.06
DCF_no_acclimation	0.398 ± 0.098	16.30 ± 2.18	0.256 ± 0.02
DCF_acclimation	0.391 ± 0.008	18.97 ± 3.03	0.261 ± 0.03
EE2_no_acclimation	0.343 ± 0.021	20.71 ± 4.62	0.264 ± 0.03
EE2_acclimation	0.335 ± 0.011	22.67 ± 2.81	0.293 ± 0.05
4NP_no_acclimation	0.299 ± 0.014	17.48 ± 5.93	0.165 ± 0.02
4NP_acclimation	0.219 ± 0.011	17.89 ± 6.23	0.298 ± 0.04

What is interesting is that the acclimation of activated sludge microorganisms did not contribute to the increase of affinity of biomass to the substrate. The values of *K*_S_ obtained in the tests, in which the acclimated biomass was applied, were on the same level or slightly higher than the corresponding values determined in the tests, in which the non-acclimated biomass was used. It was most probably connected with the fact that the concentrations of micropollutants were relatively low and did not cause the selective pressure required to change the composition of activated sludge biomass. It is also possible that the acclimation should have been performed longer. The values of maximum specific growth rate also indicated that the acclimation did not significantly influence on the growth kinetics of activated sludge biomass. The values of *μ*_max_ were on the similar level irrespective of the fact, whether acclimated or non-acclimated biomass was used in the OUR experiments (Table 1). For example, in the test with EE2, the mean *μ*_max_ values were respectively 0.343 and 0.335 h^−1^ for EE2 non-acclimated and acclimated biomass.

All determined values of *μ*_max_ fit into the range of maximum specific growth rates found for heterotrophic organisms of activated sludge treating municipal wastewater (Hauduc 2011). Generally, maximum specific growth rates were higher in the tests with micropollutants than in the control tests. Similar phenomenon was also observed in the case of OUR tests with other chemicals like imidazolium ionic liquids (Liwarska-Bizukojc et al. 2014). It is most probably associated with the elevated amount of available carbon source that favours the faster growing bacteria.

Yield coefficients of heterotrophic biomass (*Y*_XS_) were relatively low, i.e., below 0.500 mg VSS mg^−1^ COD, and their mean values varied from 0.165 to 0.298 mg VSS mg^−1^ COD (Table 1). The acclimation of activated sludge to the substrate containing the relevant micropollutant caused the increase of these yields. It indicated that the acclimation positively influenced on the synthesis of activated sludge biomass. In the case of 4NP, the positive effect of acclimation was the most significant as the value of *Y*_XS_ increased from 0.165 to 0.298 mg VSS mg^−1^ COD (Table 1).

## Conclusions

None of the tested micropollutants reduces by half the biochemical activity of microorganisms (neither the activated sludge mixed cultures nor the pure culture of *E. coli*). DCF inhibits the respiration activity of biomass to the highest extent, followed by 4NP and EE2, respectively.

The inhibitory effect of DCF on activated sludge biomass makes this compound less susceptible to biodegradation processes than EE2 and 4NP. At the same time, EE2 and 4NP are biodegradable; however, they cannot be classified as readily biodegradable compounds.

The presence of micropollutants studied in wastewater at the concentration of 10 μg L^−1^ decreases the affinity of biomass to substrate (*K*_S_), but it does not inhibit the growth of heterotrophic biomass (*μ*_max_). The acclimation of biomass to the relevant micropollutant does not influence on the kinetic parameters of biomass growth; however, it causes the increase of the yield coefficient for heterotrophic biomass (*Y*_XS_).
